# The Role of Situational Factors in Child-to-Parent Abuse: Implications for Assessment, Management, and Intervention

**DOI:** 10.1177/0306624X231159895

**Published:** 2023-03-15

**Authors:** Abigail Sheed, Natasha Maharaj, Melanie Simmons, Nina Papalia, Troy McEwan

**Affiliations:** 1 Swinburne University of Technology and Forensicare, Alphington, VIC, Australia; 2 Court Services Victoria, Melbourne, Australia; 3 University of Kent, Canterbury, UK

**Keywords:** family violence, youth, child-to-parent abuse, situation

## Abstract

Situational factors are relevant to the initiation and maintenance of violent behavior yet are infrequently examined in relation to family violence. Content analysis was used to conduct an inductive thematic analysis of police narratives to identify and quantify the occurrence of situational factors among Australian young people (10–24 years) reported to police for using violence toward a parent (*n* = 82). Descriptive information about demographics (e.g., age and sex), background characteristics (e.g., victimization history, employment/school issues, mental health issues, and neurodevelopmental conditions), and features of the index incident (e.g., type of aggression) were also extracted from police records. Interpersonal conflict and parental limit-setting were the most common situational antecedents of child-to-parent abuse, with additional situational factors including use of weapons, role of third parties, mental health concerns, and substance abuse issues. Families experiencing child-to-parent abuse showed heightened levels of intrafamilial violence and neurodevelopmental conditions. Implications for risk assessment, management, and intervention are discussed.

Situational characteristics surrounding violent events are central to theories and models explaining aggression and violence in close relationships, such as the General Aggression Model (Anderson & Bushman, 2002), I^3^ Theory (Slotter & Finkel, 2011), and the Family Violence Event Process Model (Stairmand et al., 2019). In these theories, situational factors are implicated in the initiation and maintenance of aggressive behavior and have the potential to either escalate or de-escalate violent incidents. The present study draws on the broader aggression and violence risk assessment literatures (e.g., Anderson & Bushman, 2002; Ogloff & Daffern, 2006) to define situational factors as features that are proximal (i.e., present within preceding 24 hours) to an aggressive/abusive event and either initiate and/or maintain aggressive behavior through their influence on cognition, affect, or arousal (e.g., intoxication, presence of weapons, and third parties).

## Situational Factors in the Child-to-Parent Abuse Literature

Child-to-parent abuse can be broadly defined to include physical, emotional, and psychological aggression enacted by a child toward their parent (Simmons et al., 2018). Bronfenbrenner’s (1979) nested ecological model of development (adapted by Dutton, 1995) has been used as a framework to integrate findings from the child-to-parent abuse literature, highlighting the interaction of factors across multiple levels of a young person’s ecology (e.g., individual, microsystem, and macrosystem levels; Simmons et al., 2018). This review of child-to-parent abuse using Bronfenbrenner’s (1979) model by Simmons et al. (2018) provides a more robust discussion of the factors and contexts surrounding child-to-parent abuse.

Additional research which may also be pertinent to examine in relation to child-to-parent abuse—particularly with reference to the role of situational factors—include Luckenbill’s (1977) work on homicide as a situated transaction, and Polk’s (1994) examination of the scenarios of masculine violence. Both Luckenbill (1977) and Polk (1994) highlight the role of dynamic interactions between the individual who engages in aggressive behavior, the victim, and their broader environment. Such interactionist perspectives are infrequently applied to the child-to-parent abuse research yet highlight the importance of extending research beyond the individual who uses abusive behavior to include their broader context.

While much of the research regarding the situational factors of child-to-parent abuse is fragmented and has been developed in a largely atheoretical manner, there is some consensus across studies as to which factors are relevant to abusive behavior by young people toward parents. These include, verbal arguments (Eckstein, 2002; Evans & Warren-Sohlberg, 1988; Oviedo, 2019; Retford, 2016; Simmons et al., 2018), parental limit-setting (e.g., discipline, denial of a young person’s request; Freeman, 2018; Simmons et al., 2018), substance use (Cottrell & Monk, 2004; Evans & Warren-Sohlberg, 1988; Freeman, 2018; Oviedo, 2019), mental health issues (Cottrell & Monk, 2004; Freeman, 2018; Laurent & Derry, 1999), and retaliation to the parent’s use of violence (Purcell et al., 2014).

These situational characteristics often occur within a broader context of past family violence (FV) and conflict (Cottrell & Monk, 2004; Freeman, 2018; Purcell et al., 2014), persistent behavioral problems (Laurent & Derry, 1999; Purcell et al., 2014), poverty (Cottrell & Monk, 2004), poor school engagement (Cottrell & Monk, 2004; Purcell et al., 2014), substance abuse (Cottrell & Monk, 2004; Purcell et al., 2014), and mental health issues (Freeman, 2018; Purcell et al., 2014). Some factors, such as substance use and mental health issues (Cottrell & Monk, 2004; Freeman, 2018; Purcell et al., 2014), have both proximal and distal influence, as they may precipitate an abusive incident (e.g., in the case of intoxication or acute mental health symptoms/distress), but also be related distally via their influence on a family environment already characterized by high stress and conflict.

While providing a useful starting point, this literature is limited in several key respects. First, most of the literature is decades old (Cottrell & Monk, 2004; Eckstein, 2002; Evans & Warren-Sohlberg, 1988; Laurent & Derry, 1999) or derived from doctoral dissertations which have not been subject to rigorous peer review (Eckstein, 2002; Oviedo, 2019; Retford, 2016).

Second, the more recent peer-reviewed studies appear to have identified situational factors for examination a-priori (Freeman, 2018), or situational factors were only briefly mentioned to contextualize other descriptive information (Purcell et al., 2014). This represents a substantial contrast to existing interactionist research conducted on other forms of violence (Luckenbill, 1977; Polk, 1994), which consider the dynamic interactions between the aggressive individual, the victim, and the features of their environment to be central features of analysis. Third, no known studies have examined situational factors related to child-to-parent abuse among young adults, despite clear evidence of child-to-parent abuse in this cohort (Snyder & McCurley, 2008). Finally, there is no peer-reviewed research applying knowledge of situational factors to the processes of youth FV risk assessment and intervention. While there is a need for more research identifying and describing the situational characteristics of child-to-parent abuse (Simmons et al., 2018), it is equally important that this information is then applied to inform risk assessment, risk management, and intervention approaches.

## The Role of Situational Factors in Risk Assessment

Reflecting their important precipitating role in aggression and violence, situational factors have been included in risk assessment instruments designed to assess risk for imminent violence in institutional settings. For example, tools such as the Dynamic Appraisal of Situational Aggression (DASA; Ogloff & Daffern, 2006) and the Broset Violence Checklist (BVC; Woods & Almvik, 2002), include situational risk factors like “irritability” and “sensitivity to perceived provocation.” Yet, situational characteristics of violent events are not often explicitly considered in risk instruments for general violence (Douglas et al., 2013) and family violence (Hilton et al., 2004; Kropp & Hart, 2015) outside of scenario planning (Johnstone & Logan, 2012) and formulation (Hart & Logan, 2011) .

Scenario planning is the process of imagining plausible future environments in which a given individual may engage in future violent behavior, while formulation refers to a clinician’s hypothesis about why an individual acted in a violent manner (Otto & Douglas, 2021). Both require clinicians to consider the role of situational characteristics of violent events (Johnstone & Logan, 2012; Otto & Douglas, 2021), yet there is little guidance as to what these factors are or how they might influence risk. Similarly, commonly used risk tools such as the HCR-20 (Douglas et al., 2013) and the Spousal Assault Risk Assessment (SARA; Kropp & Hart, 2015) ask clinicians to make judgements about the imminence of risk, however there is a lack of information about what factors relate to imminence.

The limited focus on situational factors in the FV field is somewhat surprising. Violence between family members invariably occurs within the context of an existing relationship. Background factors (e.g., previous victimization, mental health concerns, school/employment issues, and dysfunctional family dynamics) often exist in such relationships and provide an indication of who is relatively more or less likely to use FV over time. Yet, it is the highly changeable situational factors (e.g., intoxication, presence of a third party, and interpersonal conflict) that are most relevant to *when* violence occurs (Vagi et al., 2013). The failure to consider situational factors has been identified as one of the most significant errors in judgement that is made when assessing and managing violence risk with youth (Borum, 2000). This suggests it is essential that the FV literature engage more with these kinds of risk factors.

## The Present Study

This study adds to the limited literature examining situational factors involved in incidents of child-to-parent abuse among young people aged 10 to 24 years. Content analysis was used to conduct an inductive thematic analysis to identify the situational factors present within incidents of child-to-parent abuse reported to police in the Australian state of Victoria. Descriptive information (i.e., demographic details, background information, and characteristics of the abusive event) were also provided to allow comparison of the sample to other studies utilising police-reported child-to-parent abuse data.

## Method

### Sample

The present study uses the terms *young person who uses FV* and *FV-user*, recognising the need to consider young people as more than their behavior, and to encourage a person-centred approach to conceptualization of youth FV. This study involved the analysis of a sample of police narratives describing incidents of child-to-parent abuse in which a young person aged between 10 and 24 years was reported to police for being abusive toward a parent. The narratives were obtained from a broader sample of 500 narratives of FV police reports, half of which involved young people (aged 10–24 years; *n* = 250) who used FV. The 500 narratives were randomly selected from a population cohort of all police-reported FV incidents in the Australian state of Victoria during the 4-month period between 1 September and 31 December 2019 (index period; *N* = 24,419), excluding cases where the FV-user’s age was missing (*n* = 358, 1.50%). Victoria Police are the sole policing agency for the Australian state of Victoria, whose jurisdiction is roughly equivalent to the geographic size of the United Kingdom, with a population of 6.63 million (66.9% of whom live in the capital city of Melbourne and 18.6% who are aged 10–24 years; Australian Bureau of Statistics [ABS], 2021).

Of the 250 narratives relating to young FV-users, 104 (41.60%) described incidents of child-to-parent abuse as the primary relationship in which abuse occurred. Of these, 22 were excluded because they were duplicate narratives (*n* = 2), the narrative was not provided to researchers (*n* = 5), large sections of the narrative were missing (which likely occurred when narratives were exported across to excel spreadsheets; *n* = 11), both the young FV-user and victim refused to provide information regarding the abusive event to police (*n* = 1), the report was incorrectly recorded as child-to-parent abuse (i.e., the narrative was recorded as child-to-parent abuse when the ages of the FV-user and victim suggested that parent-to-child abuse (i.e., child maltreatment) had in fact occurred; *n* = 2), or the victim and FV-user were misidentified multiple times throughout narrative (*n* = 1). This resulted in a final analytic sample of 82 child-to-parent abuse police narratives.

### Data Source

The data used in this study were drawn from Victoria Police’s Law Enforcement Assistance Program (LEAP) database, which records all contacts between the police and the public in Victoria, including cautions, charges, victims, and FV incidents. All incidents of reported FV are recorded by Victoria Police as a matter of policy, regardless of whether an individual was charged with an offence. Victoria Police determine whether to record a FV incident, using the Family Violence Protection Act 2008, which defines FV as:

Behaviour by a person towards a family member of that person if that behaviour is physically or sexually abusive; or is emotionally or psychologically abusive; or is economically abusive; or is threatening; or coercive; or in any other way controls or dominates the family member and causes that family member to fear for the safety and wellbeing of that family member or another person (Family Violence Protection Act, 2008, s.5).

Child-to-parent abuse is a specifical relational form of family violence in which a young person aged 10 to 24 years old engages in abusive behavior toward a parent or step-parent. Abusive behavior by young people is defined in the present study with reference to the behaviors identified in the Family Violence Protection Act 2008.

Not all forms of FV identified under the Act constitute a criminal offence (e.g., no specific charges are associated with psychological abuse or coercion in Victoria at the time of publication). In Victoria, only half (*n* = 47,468, 50.8%) of all FV incidents between July 2020 and June 2021 involved a criminal offence for which charges were laid (Crime Statistics Agency, 2021).

Whenever Victoria Police members respond to an incident of FV, they are required to complete a FV report, which involves recording characteristics of the incident, the victim, the person using FV, and their relationship. This FV report also contains 39 risk factors associated with future FV or lethal FV incidents (McEwan et al., 2019), in addition to other demographic and background information on victims and FV-users.

Police narratives pertaining to FV reports are completed by the responding police officer anytime they attend a FV incident. Police officers complete FV narratives using information from the young FV-user, the victim, any third parties, and any relevant prior police records. There is no template or formal structure prescribed for responding officers when completing a narrative of a FV report, meaning the recording of situational factors is not necessarily consistent across narratives. Given this, situational factors identified in the present study reflect the proportion of times police officers mentioned the presence of a given factor, not the proportion of times it was present.

### Variable Definitions

#### Demographic and Background Characteristics

Descriptive data on demographic details, background information, and characteristics of the abusive event is provided to allow comparison of the sample to other studies of officially-recorded (i.e., administrative) child-to-parent abuse data. As this information was not always routinely provided in police narratives, it was obtained from a combination of risk factor items in the police FV reports and corresponding police narratives. Variables in the present study included: sex and age of the young FV-user and victimized parent; location of the incident (i.e., private residential or other location such as general public premises or school); socio-economic status (SES); whether the young person had prior police contact for non-FV offending or been abusive across more than one type of relationship; prior police-reported FV victimization; types of abusive behavior used by young people at the index incident; mental health issues; substance abuse issues; financial issues; and neurodevelopmental conditions.

An approximation of socioeconomic status (SES) was coded from the young person’s postcode recorded on the FV report using the corresponding decile of the ABS (2018) Index of Relative Socio-Economic Advantage and Disadvantage (IRSAD; using Victoria-specific rankings). Three SES groups were created, with low SES comprising those in the lowest 20% SES (deciles one and two), middle SES comprising the 60% in deciles 3 to 8, and high SES being those in deciles 9 and 10.

The types of abusive behavior used by young people were extracted from FV reports, while examples of the various forms of physical abuse (e.g., punching and kicking) were drawn from police narratives. Physical abuse, verbal abuse, and property damage were coded dichotomously (i.e., present or absent) and were not mutually exclusive.

Background information was obtained from a combination of risk factor items in the police FV reports and corresponding police narratives. History of police-reported victimization, including parent-to-child abuse, for young FV-users was coded dichotomously (i.e., yes or no) and represents whether a young person had ever been identified as the victim of a police-reported FV event at the time of index incident. Abusive behavior by a young person across multiple relationships was also coded dichotomously (i.e., yes or no) to show whether a young person had ever (i.e., including both the index incident and past incidents) been reported to police for using FV within two or more different types of relationships (child-to-parent abuse, intimate partner abuse, sibling abuse, other family abuse).

Variables related to the presence of mental health issues, substance abuse, financial issues, and unemployment/school truancy were recorded dichotomously based on presence, and were primarily ascertained using police questioning and discretion (i.e., asking the young person or victim if they use substances, noticing the young person appears substance affected at the time of the incident). Young people and victims did not require a formal diagnosis to be identified as experiencing mental health or substance abuse difficulties. As a result, the prevalence of these issues in the present study is likely to represent relatively gross estimates. Neurodevelopmental conditions (i.e. autism spectrum condition, attention deficit hyperactivity disorder, and intellectual disability) and types of mental health diagnoses were identified solely from police narratives.

#### Situational Factors

Information pertaining to situational factors was extracted solely from police narratives. Situational factors were defined with reference to the violence and aggression literature (Anderson & Bushman, 2002; Ogloff & Daffern, 2006) and included those features which were deemed proximal (present within the preceding 24 hours) to the abusive event and which initiated and/or maintained aggressive behavior by influencing cognition, affect, or arousal. These may include characteristics of the physical environment, characteristics of interpersonal interactions, presence of third parties, substance use around the time of the incident, and the presence of weapons, among others. Further detail about the analysis of narratives to identify situational factors is provided in the Data Analysis section.

### Procedure

All study data (i.e., FV reports and police narratives) were extracted from the LEAP database by authorized Victoria Police staff who were able to link person-level FV reports to the corresponding police narratives using unique numerical identifiers. Identifying details were removed before the data were provided to researchers for analysis.

#### Approvals and Ethics Clearances

The study was approved by the Swinburne University Human Research Ethics Committee (SUHREC: November 30, 2020, reference: 20204231-5617) and the Victoria Police Research Coordinating Committee (Project 968).

#### Data Analysis

Data analysis occurred in two steps. First, descriptive statistics summarising case demographic characteristics, background information, and features of the index FV incident were computed for the sample of 82 police narratives. To gauge sample representativeness, case characteristics (i.e., child/parent sex and age, socioeconomic status, and incident location) were also reported for the broader population of child-to-parent FV incidents from which police narratives were drawn.

Second, police narratives were analyzed using content analysis methodology to identify and quantify the prevalence of situational themes relevant to the use of child-to-parent abuse by young people. Themes were generated through inductive thematic analysis using a mix of manual coding and NVivo software. Police narratives were initially analyzed through multiple readings by the lead author (A.S) and were read line-by-line to identify and label individual meaning units. A.S. regularly consulted with N.M. and T.M. regarding the accuracy of coding. Once themes and subthemes were finalized, A.S. provided coding training to author M.S. for the purposes of determining inter-rater reliability of situational factors to ensure consistent frequency estimates. M.S. independently coded the situational factors from nine randomly selected narratives at the subtheme level. The presence of subthemes was agreed upon in 70.83% of cases (7 out of a total of 24 subthemes misidentified), while interrater reliability at the theme level was identified as 84.21% (3 out of a total of 19 themes misidentified).

## Results

### Descriptive and Background Characteristics

Table 1 displays the demographic characteristics of the sample relative to the broader population of youth (i.e., FV-user was aged 10–24 years at the time of the index incident) child-to-parent incident reports from which the sample was derived. Young people who used child-to-parent abuse were primarily males who engaged in abusive behavior toward their mothers at a private residence. Those who were reported to police for child-to-parent abuse were most commonly aged 15 to 19 years old and resided in a low socioeconomic status area.

**Table 1.** Demographic Characteristics of All Cases of Child-to-Parent Abuse in the Total Dataset (*N* = 2,029) and Those in the Narrative Dataset (*n* = 82) for Youth Aged 10 to 24 years.

*n*	Total dataset of CPA *N* (%)	Narrative dataset *n* (%)
	2,029	82
Characteristics of young person		
Sex		
Male	1,357 (66.88)	57 (69.51)
Female	672 (33.12)	25 (30.49)
Age		
10–14 years	400 (19.71)	17 (20.73)
15–19 years	979 (48.25)	43 (52.44)
20–24 years	650 (32.04)	22 (26.83)
Lowest 20% SES	616 (30.36)	24 (29.27)
Prior non-family violence offending		
Generalist	955 (47.07)	36 (43.90)
Family-only	1,074 (52.93)	46 (56.10)
Characteristics of victimized parent		
Sex		
Female	1,546 (76.20)	60 (73.17)
Male	480 (23.66)	22 (26.83)
Age (*M*, *SD*)	47.12 (8.31)	47.55 (7.18)
Characteristics of the incident		
Location of index incident		
Private residential	1,860 (91.67)	74 (90.24)
Other location^a^	169 (8.33)	8 (9.76)

*Note*. CPA = child-to-parent abuse.

a Other location refers to any non-private residence (e.g., general public premises, educational premises, legal premises, and public housing premises).

The broader background characteristics of the 82 police-reported child-to-parent abuse cases were also examined. Twenty-two percent (*n* = 18) of young people had been previously reported to police for abusive behavior toward a different family member. More than one third (*n* = 28, 34.15%) of young people had a history of police-reported FV victimization, with more than half (*n* = 15, 53.57%) of those young people being victims of police-reported parent-to-child abuse. Thus, incidents of police-reported child-to-parent abuse often occurred in the context of elevated levels of intrafamilial conflict and violence.

Mental health issues were common among both young people and victimized parents, with over half of all young people (*n* = 47, 57.32%), and more than one in five parents (*n* = 19, 23.17%) having such issues recorded by police. Anxiety and trauma-related conditions (*n* = 8) were most commonly reported among young people, followed by mood disorders (*n* = 5), and psychotic disorders (*n* = 2). Further, more than 1 in 10 (*n* = 11, 13.41%) young FV-users were identified in police narratives as having a neurodevelopmental condition (i.e., attention deficit hyperactivity disorder (ADHD), autism spectrum condition, or learning disability).

More than one third (*n* = 29, 35.37%) of young people were reported to have substance abuse issues, with 28.05% (*n* = 23) identified by police as likely to be substance-affected at the time of the index incident. Cannabis and alcohol were identified in police narratives as the most frequently used substances by young people, with methamphetamines and benzodiazepines also mentioned. Nearly half of youth experienced schooling or employment issues (*n* = 14, 17.07%), and almost one quarter (*n* = 19, 23.17%) of families were identified as experiencing financial issues.

### Characteristics of the Abusive Event

Most incidents (*n* = 74, 90.24%) occurred at a private residence, such as the family home, while nearly 1 in 10 (*n* = 8, 9.76%) occurred in public, such as at a school, police station, or on the street. Approximately one in five (*n* = 18, 21.95%) incidents involved the young person engaging in physical abuse, including pushing, kicking, slapping, and punching. The most severe forms of violence identified in the narratives included strangulation and attempting to set the victimized parent on fire. Nearly three quarters (*n* = 65, 79.27%) of incidents involved verbal abuse, and 17.07% (*n* = 14) involved property damage. Approximately 1 in 10 (*n* = 8, 9.76%) FV reports noted that no violent or otherwise abusive behavior was identified by police.

### Situational Characteristics at the Time of the Index Family Violence Incident

Six overarching themes were derived from the content analysis of situational factors within police narratives. Interpersonal conflict (e.g., arguments) and parental limit-setting (e.g., making requests/placing constraints behavior) were the most common immediate antecedents of child-to-parent abuse. Additional situational factors which were identified included weapons, role of third parties, mental health issues, and substance abuse issues. Subthemes are underlined, while quotes are italicized.

#### Interpersonal Conflict

Interpersonal conflict was unsurprisingly a major theme, occurring in 73.29% (*n* = 61) of incidents. Conflict both preceded and maintained abusive events. A range of subthemes were observed under the umbrella of interpersonal conflict, which are described below.

Verbal arguments preceded over half (53.66%, *n* = 44) of all police-reported child-to-parent abuse incidents and were the most common form of interpersonal conflict. Arguments often occurred in the context of parental limit-setting, or an argument between the young person and a third party (e.g., a sibling). In a small number of cases, arguments were noted to persist over a prolonged period (i.e., hours), with an eventual escalation in abusive behavior, or parents calling police for support with de-escalation.

*An argument has occurred this evening when [the victim] has had a go [i.e., yelled at] at the [young person] over some dirty dishes. [The victim] was out the front of her van when [the] argument has become physical with [the young person] pushing her forcefully with both hands caused her to fall backwards.*

A less common but observable subtheme involved parents intervening in existing conflict (*n* = 4, 4.88%), often between the young person and their siblings, but occasionally between the young person and their intimate partner. Intervention by the victimized parent typically occurred in the context of a young person exhibiting abusive behavior toward another individual. Often the young person diverted their attention from the third party and became either verbally or physically abusive toward the intervening parent. Rarely, the young person intervened in conflict between the parent and a third party and became abusive.

*On this occasion, the [young person] became verbally abusive towards her youngest brother. The [young person] began kicking him, the [victim] attempted to remove her, which resulted in the [young person] turning her attention to [the victim]. The [young person] began kicking the [victim].*

Parents were also observed attempting to de-escalate the young person’s behavior, precipitating abuse or further abuse. Some parents attempted to calm the young person down verbally or by not responding to them, while others would attempt to physically restrain the young person, either seeking to calm them down or due to fear of physical assault.

*The [victim] stated that the [young person] had an argument with his sister, who had then left the premises. The [victim] stated that she had pinned [the young person] to the ground outside to keep him calm.*

Attempts at de-escalation were observed to typically be ineffective, however this is likely the result of sampling bias. Those situations in which de-escalation attempts were successful would be unlikely to involve police intervention.

In some cases (15.85%, *n* = 13), retaliation by the victimized parent was observed, often in the context of the young person’s persistent abusive behavior. Physical and verbal forms of retaliation were identified within police narratives, with both forms typically associated with an escalation in abuse by the young person. This is not to say that the actions of victimized parents are to blame for their experiences of abuse, but rather to highlight that retaliation is a situational factor which may be associated with greater violence risk. Physical forms of retaliation by mothers and fathers included pushing the young person, grabbing the young person’s clothing or hands, arming oneself with a weapon, or punching back.

*The young person has then entered the kitchen and pulled out all the cupboard drawers and the cutlery has gone all over the floor. The [victim] then went inside and said to the [young person] “is that all you’ve got?”, which has infuriated the [young person] . . . a push and shove has then occurred . . . and the [victim] phoned police.*

#### Limit-Setting

Issues pertaining to limit-setting were observed in half (50.00%, *n* = 41) of all narratives and was noted to be a key situational antecedent of child-to-parent abuse among those with neurodevelopmental conditions. Limit-setting typically involved the parent making a request or placing constraints on behavior of the young person. This included limiting a young person’s use of technology, making a request of the young person, intervening to stop interpersonal conflict or abusive behavior, or preventing a young person from leaving the house due to a curfew or as a form of punishment.

*The [victim] stood at the [young person’s] door and demanded she clean her room up . . . the argument got heated between the [young person] and the [victim], with the [young person] attempting to push the [victim] out the bedroom to close the door.*

Limit-setting frequently precipitated interpersonal conflict and was most often carried out by the victimized parent. Denial of the young person’s requests by the victimized parent was associated with high levels of interpersonal conflict and often precipitated verbal abuse.

Limit-setting could also precipitate or escalate abuse when the young person was asked to leave because of their behavior.

*The [young person] got extremely agitated causing things to get tense so the [victim] told the [young person] to pack his stuff and leave. [The young person] threw his laptop through the window, smashing the window and punching the wall causing a hole.*

Parents were observed to employ police as disciplinarians in situations where it appeared they felt incapable of setting limits with the young person, or where there had been persistent abuse and the parent felt unable to manage the behavior any longer. Alternatively, parents were also observed to request police involvement where no abusive behavior had in fact occurred but where they wanted support from the police to discipline or effectively communicate with the young person.

*[The victim] stated [no] concerns for safety and called police as she wanted police to speak to the [young person] about getting the tattoo and the [young person’s] drug use.*

#### Substance Use

Substance use was described in 24.39% (*n* = 20) of police narratives as a situational characteristic, with a high level of co-occurring school/employment issues and mental health issues within these narratives. Substance use was identified as a source of interpersonal conflict and/or the focus of parental limit-setting. For example, in some instances, parents refused to provide the young person with money for fear it would be used to buy substances, while others raised concerns regarding a young person’s friends due to their substance use.

*The [victim] had told the [young person] that she can’t have friends around due to their drug usage. This has caused the [young person] to become angry . . . The [young person] grabbed a knife from the kitchen and put it through one of the blinds in the dining area.*

The effects of intoxication or withdrawal from substances featured in 12.20% (*n* = 10) of narratives. This differed substantially from what was recorded in FV reports, which identified more than one quarter (28.05%) of all youth as likely being substance-affected at the time of the index incident. Young people who were intoxicated at the time of the index incident were noted to experience broader issues with substance abuse which frequently occurred in the context of ongoing abusive behavior and housing instability.

Rarely, parental substance use or intoxication was observed. This typically resulted in a reversal of the parent-child dynamic in which the young person attempted to set limits on their parent’s behavior, leading the parent to call police.

#### Presence/Involvement of Third Party

Third parties were commonly identified as present in incidents (*n* = 33, 40.24%) and included the young person’s siblings or other parent, friends, intimate partners, other family members, or strangers. One in ten (*n* = 8, 9.76%) young people were noted to have displayed abusive behavior toward multiple family members during the index incident. For example, the incident may have started with conflict between the young person and sibling, following which the parent intervened and became the primary victim recorded by police.

*[The young person] and [the victim] have been arguing over the [young person] being on his iPad too much and [the young person] has then become verbally abusive towards her and brother of [the young person].*

In cases where third parties were present, they were typically involved in de-escalating conflict, including restraining the young person to prevent further abusive behavior. However, at times, the presence of a third party escalated a situation as their presence appeared to exacerbate the young person’s emotional response to other situational variables (e.g., parental limit-setting).

*The [victim] stood at the [young person’s] door and demanded she clean her room and pick up clothing . . . The [young person] was embarrassed as her friend was present when her mother was telling her off. This got the [young person] upset so she pushed the [victim].*

Limit-setting by third parties was observed in a minority of narratives (*n* = 7). Typically, this involved the third party/parties attempting to physically restrain or remove the young person to prevent violence, but also included asking the young person to leave or preventing them from entering the home. Rarely (*n* = 3), victimized parents explicitly called a third party for support in managing the young person’s behavior.

#### Weapons

Weapons featured infrequently in police-reported incidents of child-to-parent abuse, with 17.07% (*n* = 14) of narratives identifying the presence of a weapon. Three young people were recorded by police as using a weapon and another three made explicit threats with the weapon (either to self or others). However, in most cases where weapons were present, they appeared to be items close at hand (e.g., knives were present if the incident occurred in the kitchen), with youth typically picking them up impulsively and quickly discarding them.

*The [young person] grabbed a metal object and walked towards the family living room however no threats were made, and the object was not used to hit anything. The [young person] then got rid of the object and walked towards his room.*

While some parents attempted to remove the weapon from the young person, others attempted to verbally de-escalate the situation, or the weapon prompted them to call police.

#### Mental Health and Threats of Self-Harm

In a minority of narratives (*n* = 4, 4.88%), young people who engaged in child-to-parent abuse were recorded as having threatened self-harm during the index incident. Threats of self-harm often occurred in response to police being called.

*The [young person] went to his room and locked the door . . . stating that he wanted to kill himself . . . [he stated he] made the threat as he didn’t want the [victim] to call police.*

Narratives rarely identified incidents as occurring in the context of poor parental mental health (*n* = 2, 2.44%) or due to a parent’s concern for the young person’s mental health (*n* = 1, 1.22%). Some young people were identified as not having taken medication prescribed for mental health concerns, leading to emotional dysregulation and parents’ perception of an elevated risk of aggression.

## Discussion

The present study sought to identify situational factors relevant to police-reported incidents of child-to-parent abuse. Interpersonal conflict (e.g., verbal arguments, parents intervening in existing conflict, and retaliation) and parental limit-setting (e.g., young person being asked to leave, enforcing house rules) were the most commonly described situational antecedents of child-to-parent abuse, which is broadly consistent with existing research from different jurisdictions, including the United States (Oviedo, 2019), Canada (Cottrell & Monk, 2004), and Australia (Freeman, 2018; Simmons et al., 2018). Substance abuse (e.g., intoxication, arguments about substances, and parental substance abuse), third party involvement, presence of weapons, and mental health issues have also been identified as additional situational antecedents of child-to-parent abuse across various jurisdictions, including France (Laurent & Derry, 1999), Canada (Cottrell & Monk, 2004), Australia (Freeman, 2018), and the United States (Evans & Warren-Sohlberg, 1988).

Situational factors interact with the broader context of an individual’s life, creating an environment in which young people with certain predisposing characteristics are more likely to engage in violent or abusive behavior. Consistent with a wealth of previous research, families reporting child-to-parent abuse in this study experienced a confluence of predisposing background factors such as elevated levels of intrafamilial violence, mental health issues, substance abuse, neurodevelopmental conditions, education/employment instability, and broader life stressors (e.g., financial issues; Calvete & Orue, 2016; Cottrell & Monk, 2004 ; Simmons et al., 2018). These frequently coalesced to create heightened levels of familial stress, which is associated with increased risk of FV (Cottrell & Monk, 2004).

The heightened level of interpersonal conflict and difficulties with parental limit-setting observed within these high-need/high-stress familial environments are often explained in the context of dysfunctional communication patterns, coercive interaction sequences, or simply a lack of parenting skills (Simmons et al., 2018). Problematic familial interactions may be compounded by underlying psychological characteristics such as the hostile attributions, justification of violence, impulsivity, and diminished capacity for interpersonal problem-solving (Contreras & Cano, 2015; Orue et al., 2021; Simmons et al., 2018) displayed by young people who engage in child-to-parent abuse. These characteristics have in turn been linked to the higher levels of victimization and FV exposure among this group of young people (Contreras & Cano, 2015; Simmons et al., 2018).

### Limitations and Future Directions

The use of police narratives as a data source presents several limitations. Narratives only capture situational factors the responding police officer thought relevant to the index incident, and so do not represent a comprehensive source of situational antecedents of youth FV events. Similarly, the quality and quantity of information contained in narratives is dependent upon the responding officer’s capacity to interview the victim, young person, and any third parties. Despite these limitations, the narratives assisted in the identification of a common set of situational characteristics associated with the use of child-to-parent abuse.

The use of police records meant that only FV incidents that were officially reported were captured, limiting generalizability of the findings to community samples. While this is an important limitation, it does not significantly impact upon the importance of these results for the purposes of risk assessment and intervention by police or referral agencies who respond to police reports. The third limitation relates to the small sample size (*n* = 82) and focus on a single type of abusive relationship (i.e., child-to-parent abuse) employed within the present study. The small sample size may have prevented the full range of situational variables relevant to child-to-parent abuse from being captured and overlooks the interconnectedness of different forms of violence.

Future research would benefit from continuing to identify situational factors related to youth FV events using self-reported measures and longitudinal designs. More attempts should be made to incorporate multiple relationships of abuse (e.g., child-to-parent abuse and intimate partner abuse) within future research, as the adult FV literature indicates the event processes may be similar across relationships (Stairmand et al., 2019).

### Implications for Practice

In light of the limitations and need for future research, the findings of the present study have several important implications for risk assessment, risk management, and therapeutic intervention. First, many young people were noted to experience multiple needs concurrently, indicating risk management and intervention approaches need to be cognisant of the role and impact of these co-occurring needs on behavior. For example, those who use FV while intoxicated are likely to have concurrent mental health issues and school/employment issues which need to be addressed alongside their substance use and FV. Second, the over-representation of neurodevelopmental conditions emphasizes the need for risk management and intervention approaches to be cognisant of this important responsivity issue.

Third, nearly 1 in 10 young people were identified as being abusive toward multiple family members at the time of the index incident, while more than one in five had ever been reported to police for being abusive across more than one type of relationship (i.e., at the index incident and at least one other incident). This highlights the importance of services adopting a whole-of-family approach to treating and managing child-to-parent abuse, such as occurs in family systems therapy (Micucci, 1995). Additionally, those engaging in risk assessment with young people and affected families must ensure they adequately consider the young person’s risk of abusing multiple family members, rather than focusing exclusively on the reported victim.

Fourth, understanding common situational antecedents of child-to-parent abuse can help clinicians with scenario planning, for which there is currently little guidance (DeMatteo et al., 2010). Identification of such factors assists in tailoring risk management strategies to the situational triggers of a young person’s behavior. This may enhance the likelihood interventions will be implemented and alter the chain of events (Rizvi & Sayers, 2020).

Scenario planning could in fact be aided by the application of functional analysis. Functional analysis provides insight into the drivers and consequences of violent behavior and helps to identify risk management and intervention targets (McGarity et al., 2021). The findings of the present study can be used to provide guidance for clinicians engaging in functional analysis and scenario planning with young people who use FV Additionally, the results provided in this study may be particularly relevant for those who engage in scenario planning but are not trained in the intricacies of functional analysis, or who work in settings where a thorough functional analysis is not possible (e.g., working with those with significant cognitive impairment and lack of time or resources).

Fifth, youth FV research may benefit from drawing on the inpatient aggression literature, which has a wealth of information on how to de-escalate, set limits, and communicate with inpatients, as well as implement appropriate risk management strategies (Maguire et al., 2019; Slaatto et al., 2021). For example, the denial of requests by staff and an unwillingness of patients to follow directions are identified in the inpatient aggression literature to be key situational antecedents of violence (Ogloff & Daffern, 2006), and appear somewhat analogous to the issues with parental limit-setting observed in the present study. Similar approaches may even be applicable to managing and intervening with other forms of youth FV, including aggression toward carers in the out-of-home care system.

## Conclusions

The findings of this study highlight the importance of considering the situational characteristics of child-to-parent abuse and their relevance to risk assessment, risk management, and intervention. Content analysis of police narratives pertaining to incidents of child-to-parent abuse highlighted elevated levels of interpersonal conflict and issues with parental limit-setting as the most common situational factors involved in this form of FV. Consistent with the extant research, child-to-parent abuse was found to occur among families experiencing a confluence of predisposing background factors, highlighting the importance of considering and addressing multiple areas of need (i.e., mental health, substance abuse, and education/employment instability) concurrently when assessing risk and intervening with affected youth and families. Additionally, this is one of few studies highlighting the importance of considering neurodevelopmental conditions as a responsivity issue when assessing and managing child-to-parent abuse. Important lessons in managing and intervening with youth FV may be derived from the inpatient aggression literature, which has a robust literature base on how to communicate, de-escalate, and set limits with individuals at heightened risk for aggression.
